# Amplification-free library preparation with SAFE Hi-C uses ligation products for deep sequencing to improve traditional Hi-C analysis

**DOI:** 10.1038/s42003-019-0519-y

**Published:** 2019-07-19

**Authors:** Longjian Niu, Wei Shen, Yingzhang Huang, Na He, Yuedong Zhang, Jialei Sun, Jing Wan, Daxin Jiang, Manyun Yang, Yu Chung Tse, Li Li, Chunhui Hou

**Affiliations:** 1 Department of Biology, Southern University of Science and Technology, 518055 Shenzhen, China; 2 Department of Biology, Nankai University, 300071 Tianjin, China; 3 Department of Bioinformatics, Huazhong Agricultural University, 430070 Wuhan, China; 4 Hubei Key Laboratory of Agricultural Bioinformatics, Huazhong Agricultural University, 430070 Wuhan, China

**Keywords:** Bioinformatics, Epigenomics, Chromatin structure

## Abstract

PCR amplification of Hi-C libraries introduces unusable duplicates and results in a biased representation of chromatin interactions. We present a simplified, fast, and economically efficient Hi-C library preparation procedure, SAFE Hi-C, which generates sufficient non-amplified ligation products for deep sequencing from 30 million *Drosophila* cells. Comprehensive analysis of the resulting data shows that amplification-free Hi-C preserves higher complexity of chromatin interaction and lowers sequencing depth for the same number of unique paired reads. For human cells which have a large genome, SAFE Hi-C recovers enough ligated fragments for direct high-throughput sequencing without amplification from as few as 250,000 cells. Comparison with published in situ Hi-C data from millions of human cells demonstrates that amplification introduces distance-dependent amplification bias, which results in an increased background noise level against genomic distance. With amplification bias avoided, SAFE Hi-C may produce a chromatin interaction network more faithfully reflecting the real three-dimensional genomic architecture.

## Abstract (web summary)

Niu, Shen, Huang et al. presents SAFE Hi-C, a Hi-C library preparation method that uses ligation products to prepare a library for deep sequencing without amplification. They show that SAFE Hi-C can build a chromatin interaction network more faithfully than traditional Hi-C by preserving higher complexity of chromatin interaction and lowering sequencing depth.

## Introduction

Hi-C is a powerful tool for mapping interaction frequencies between chromatin fragments in a genome-wide and quantitative manner^1^. It compares the number of ligation events between each fragment pair in a large population of cells and thereby allows the identification of various genome structural features, including compartments, topologically associated domains (TADs), and loops^1–5^. The main prerequisite for high-quality Hi-C analysis is the accurate quantification of chromatin interaction frequency. The amount of DNA obtained in a typical Hi-C experiment is assumed to be insufficient for direct high-throughput sequencing. Thus, PCR amplification is a default step in Hi-C-related experiments^6^ to guarantee sequencing primer addition and to produce a sufficient amount of DNA for sequencing, especially for Hi-C experiments involving single or low number of cell^7–18^.

The three-dimensional (3D) nature of Hi-C deems sequencing depth and library complexity are two critical variables in evaluating the achievable resolution of Hi-C experiments, given a range of fragment sizes predetermined by the choice of restriction enzyme for a specific genome. Currently, many biological replicates and multiple rounds of PCR amplifications are required for high-resolution genome architecture analysis in order to generate sufficient DNA with high enough complexity to represent the global chromatin interaction diversity within a cell population. Although universal primers are used, PCR amplification introduces duplicates and may skew Hi-C library composition, which may not be fully corrected by normalization methods^19–21^. Efficient recovery of enough ligated fragments for direct high-throughput sequencing is important for the accurate characterization and understanding of the 3D genome architecture and its functional role in transcription regulation, replication, genome stability, and other critical biological activities happening at the chromatin level. However, whether PCR amplification is really necessary and to what extent it changes the composition of Hi-C library had not been systematically evaluated.

Here we present SAFE Hi-C, a *s*implified, *a*mplification-*f*ree, and economically *e*fficient process, in which paired reads generated by independent ligation events were saved. We tested this method on 30 million *Drosophila* S2 and 250 thousand human K562 cells. Comparison to traditional in situ Hi-C revealed that SAFE Hi-C effectively reduced distance-dependent bias in chromatin interaction frequency, increased resolution, and improved analysis reliability. Taken together, our results suggest that it is advantageous to avoid PCR amplification, thus improving the quality of Hi-C analysis by SAFE Hi-C.

## Results

### SAFE Hi-C library preparation and sequencing

**Fig. 1:**
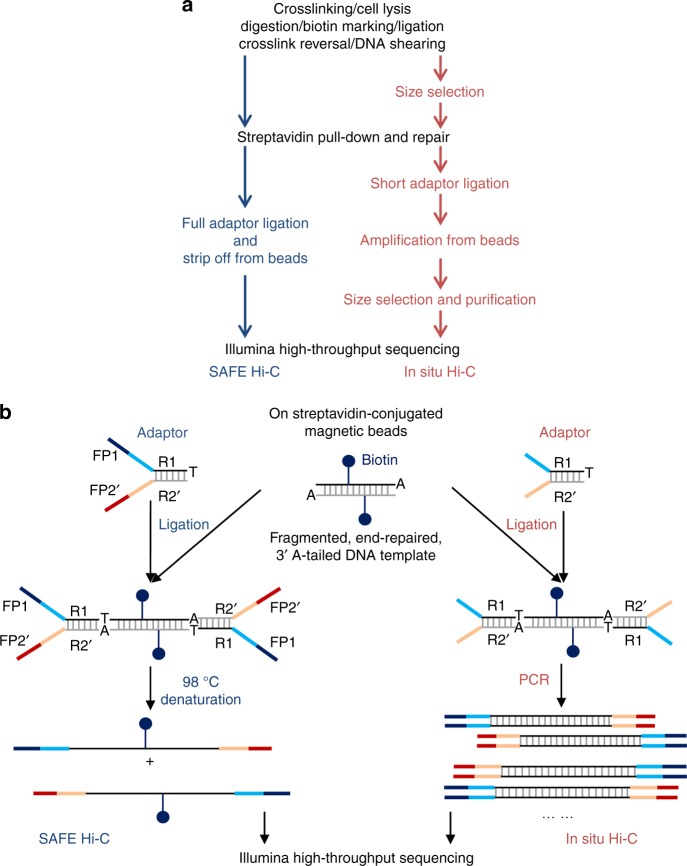
Procedures of SAFE (*s*implified, *a*mplification-*f*ree, and economically *e*fficient process) Hi-C and traditional in situ Hi-C. **a** Side-by-side comparison of SAFE Hi-C and in situ Hi-C procedures. The black text shows shared steps in both methods, blue and red texts correspond to steps specific for SAFE Hi-C and in situ Hi-C, respectively. **b** In the preparation of both in situ Hi-C and SAFE Hi-C libraries, partially complementary adapters with a 3′ thymine (T) overhang were ligated to repaired and 3′ adenine (A)-tailed DNA fragments having been captured on streptavidin beads. The sequence of adaptors is listed in Supplementary Table 1

To determine how many biotin-labelled ligation events can be captured by streptavidin-conjugated beads, we carried out two SAFE Hi-C experiments as biological replicates on 30 million *Drosophila* S2 cells using *Dpn*II (Fig. 1a). We stripped off Hi-C ligation products from beads after the addition of sequencing primers^22^ (Fig. 1b and Supplementary Table 1). Quantification showed that each SAFE Hi-C experiment recovered around 100 ng of ligated DNA, which roughly equals to 50 μl of 12 nM single-strand DNA with a size around 500 bases, enough for at least 10 lanes of sequencing on the Illumina HiSeq X10 platform. We also conducted traditional in situ Hi-C experiments in replicates for comparison and amplified Hi-C ligation products from diluted beads using different numbers of PCR amplification cycles (4, 8, 12, 16, and 20) to produce similar amounts of DNA as in the SAFE Hi-C experiments.

**Fig. 2:**
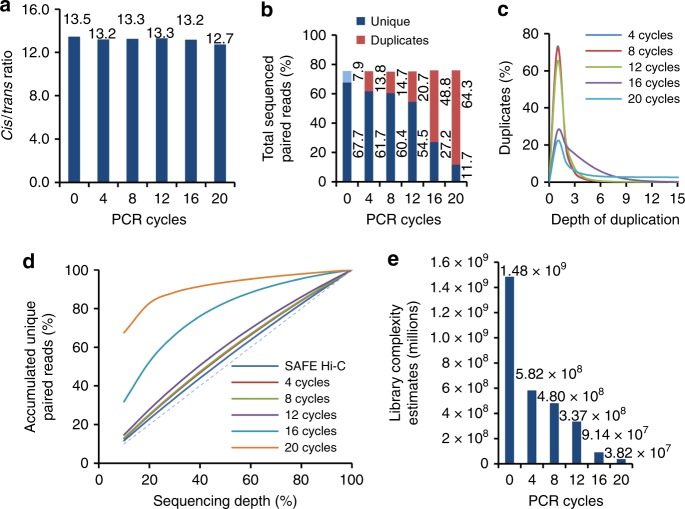
Amplification increases PCR duplicates and reduces Hi-C library complexity of the *Drosophila* genome. **a** *Cis* paired reads were uniquely mapped on the same chromosome, and *trans* paired reads were mapped on different chromosomes. SAFE (simplified, amplification-free, and economically efficient process) Hi-C is referred to as 0 PCR cycle in figures. **b** Percentage of unique paired reads and duplicates that can be aligned in the total sequenced paired reads. The light blue bar shows the duplicates in SAFE Hi-C libraries, which we kept as unique as no amplification was involved in the library preparation process. **c** Percentage of ligates duplicated at different depths introduced by PCR amplification. **d** Accumulated percentage of unique paired reads against the percentage of sequencing depth. **e** Library complexity estimates. For SAFE Hi-C, the complexity was estimated as described in Methods

All libraries were sequenced on an Illumina HiSeq X10 instrument and aligned to the reference genome using bowtie 2.0^23^. The length of sequenced fragments in all Hi-C libraries ranged from 200 to 750 bp, and peaked around 370 bp (Supplementary Fig. 1), consistent with the fact that the median length of *Dpn*II fragments is 194 bp. Global chromatin interaction frequencies were highly correlated between biological replicates and between different pairs of libraries, with the lowest stratum-adjusted correlation coefficient (SCC) of 0.994 (Supplementary Fig. 2). We combined biological replicates and obtained 338, 246, 220, 232, 238, and 248 million aligned paired reads (Supplementary Table 2). Datasets were normalized as described^5,24^ for further analysis. The ratio of *cis-* and *trans*-unique paired reads, generally considered as a proxy indicator of the quality of a Hi-C library, was 13.5 for SAFE Hi-C and approximately the same for amplified libraries (Fig. 2a). After PCR amplification, fragment pairs with lower (<~42%) or higher (>~42%) GC content were significantly under- or over-represented compared to SAFE Hi-C, respectively (*p* < 10^−40^, Mann–Whitney *U* test, Supplementary Fig. 3).

After successfully applying SAFE Hi-C on 30 million *Drosophila* cells whose chromatin content is roughly equal to 1 million human cells, next we tested the lowest number of human cells needed for SAFE Hi-C. Two hundred and fifty thousand and 100 thousand human K562 cells were used. SAFE Hi-C was successful only with 250 thousand cells from which we recovered 15 μl of 4 nM single-strand DNA with a size around 575 bases. This is about 1/10 of the DNA recovered from 30 million *Drosophila* S2 cells. This could be caused by the higher rate of DNA loss when small amount of starting material was used for Hi-C. The human K562 SAFE Hi-C library was sequenced on an Illumina HiSeq X10 platform and 121 million paired reads were generated, out of which 106 million (87.4%) paired reads aligned successfully to the human reference genome (Supplementary Table 3). A similar number of chromatin interactions from in situ Hi-C on K562 cells previously published by Rao et al.^5^ was downloaded and used for comparison.

### SAFE Hi-C avoids removal of PCR duplicates

PCR amplification introduces duplicates to the Hi-C library, which lowers the percentage of unique paired reads. For SAFE Hi-C, we kept duplicates because they were generated by independent ligation of fragment pairs of the same sequences and no amplification was involved. Differently, optical duplicates were generated through the DNA cluster generation process on the Illumina sequencing machine. Optical duplicates account for <1% of the total sequenced paired reads (Supplementary Table 2) and were excluded in further analysis.

About 8% of total mapped paired reads were duplicates in *Drosophila* S2 SAFE Hi-C libraries (Fig. 2b, light blue bar) and were kept for Hi-C analysis. However, for amplified libraries, duplicates from independent ligations cannot be distinguished from those introduced by PCR amplification, thus all were considered arising from single ligation event. As expected, the proportion of PCR duplicates positively correlated with the number of amplification cycles (Fig. 2b, red bar). The percentage of duplicates increased to 14%, 15%, 21%, 49%, and 64% after 4, 8, 12, 16, and 20 cycles of amplification, respectively (Fig. 2b, red bar). Correspondingly, the percentage of non-duplicate paired reads decreased dramatically as amplification cycles increased, especially after 16 and 20 cycles (Fig. 2b, dark blue bar). We also calculated the percentages of duplicates and of valid paired reads for all mappable ligated fragments (Supplementary Fig. 4). PCR duplicate depth analysis showed that most duplicated ligates had two copies in all libraries (Fig. 2c), and the percentage of ligates of higher duplication increased considerably after 16 and 20 cycles of amplification (Fig. 2c).

### SAFE Hi-C increases library complexity

For *Drosophila* S2 SAFE Hi-C libraries, the percentage of unique paired reads correlated almost linearly with sequencing depth (Fig. 2d), suggesting that the library complexity was far from exhausted at current sequencing depth. After amplification, ligates of same sequence considered as PCR duplicates increased dramatically (Fig. 2b). Consistently, the estimated library complexity dropped sharply from 1.5 billion for SAFE Hi-C libraries to 0.58 billion for in situ Hi-C libraries after only four cycles of amplification (Fig. 2e).

### Amplification bias is genomic distance dependent

**Fig. 3:**
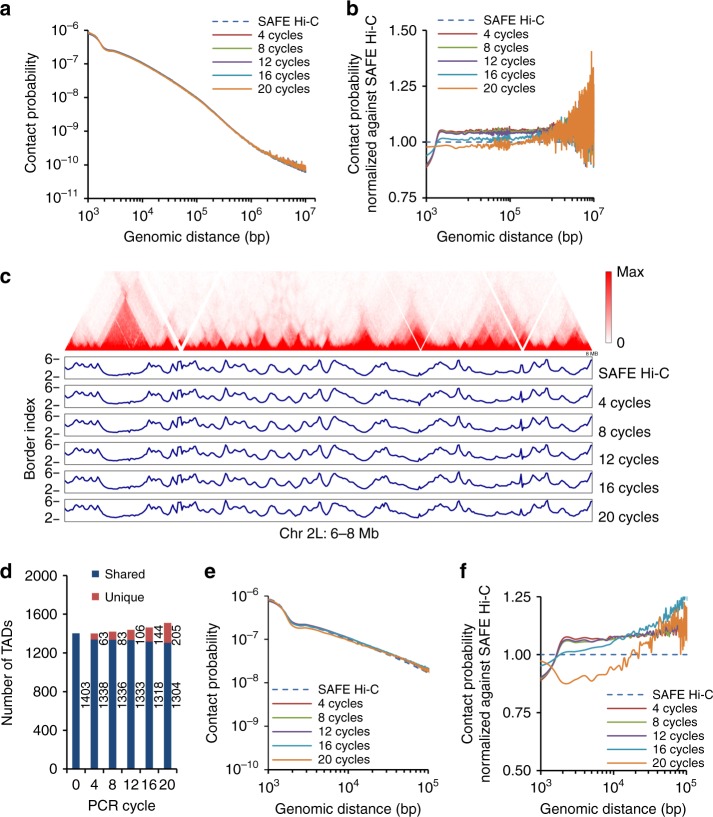
Distance-related amplification bias and topologically associated domain (TAD) identification in *Drosophila* genome. **a** Chromatin interaction frequency as a function of genomic distance averaged across the *Drosophila* genome. **b** Average chromatin interaction frequency across the genome normalized against SAFE (simplified, amplification-free, and economically efficient process) Hi-C for the *Drosophila* genome. **c** Hi-C interaction heatmap for the region of chromosome 2 L from 6 to 8 Mb is shown. Fluctuation pattern of border index is shown below heatmap for SAFE Hi-C and amplified Hi-C. **d** Dark blue bars show the number of TADs shared with SAFE Hi-C; red bars show the number of TADs uniquely found for amplified Hi-C libraries. **e** Chromatin interaction frequency averaged within the TADs was plotted as a function of genomic distance. **f** Average chromatin interaction frequency within the TADs was normalized against SAFE Hi-C

Chromatin interaction frequency is inversely correlated with genomic distance. Amplification resulted in a moderate change on the decaying pattern of chromatin interactions at any genomic distance for traditional in situ Hi-Cs on *Drosophila* S2 cells (Fig. 3a). After normalization against SAFE Hi-C, we found that the relative chromatin interaction frequency started lower (~0.9) at 1 kb and became higher beyond 3 kb for most amplified libraries of *Drosophila* S2 cells, except for library amplified for 20 cycles (Fig. 3b), suggesting that short-distance ligations were generally underrepresented after even only four cycles of amplification.

**Fig. 4:**
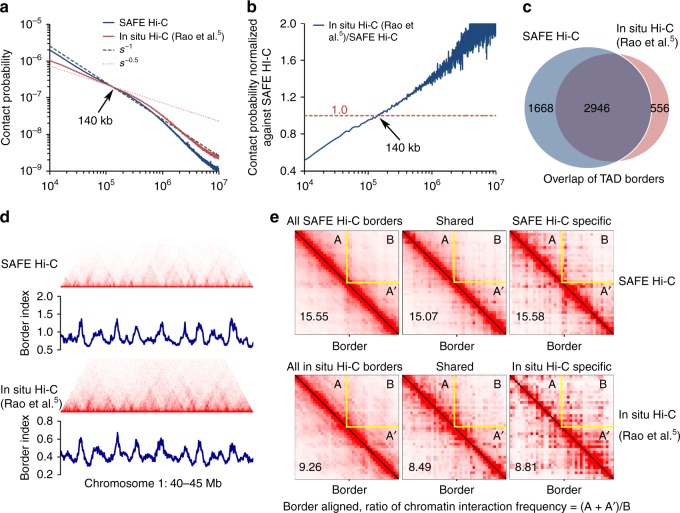
SAFE (simplified, amplification-free, and economically efficient process) Hi-C on 250 thousand human K562 cells. **a** Comparison of chromatin interaction frequency against the genomic distance between SAFE Hi-C (blue line) and in situ Hi-C (red line). *s*^−1^ (black dashed line) and *s*^−0.5^ (red dotted line) represent the predicted fractal globule and mitotic states, respectively. The turning point of chromatin interactions on the decaying curves for SAFE Hi-C and in situ Hi-C is indicated by an arrowhead. **b** Average chromatin interaction frequency of in situ Hi-C was normalized against SAFE Hi-C across the genome. The crossing point is shown by an arrowhead. **c** Venn diagram shows the overlap between topologically associated domains (TADs) identified for SAFE Hi-C and in situ Hi-C. **d** Chromatin contact heatmap and border index comparison between SAFE Hi-C and in situ Hi-C for a region in chromosome 1 from 40 to 45 Mb. **e** Borders were aligned for all, shared, and specific TADs. The chromatin contact frequency in flanking TADs (A + A′) was divided by that of the inter-TADs (B), the ratios of (A + A′)/B for aggregated borders are shown at the bottom left in each heatmap

In comparison to *Drosophila S2*, amplification introduced more obvious biases for the human genome. Compared to published in situ Hi-C on human K562 cells, chromatin interaction frequency of SAFE Hi-C decayed at a rate much closer to the predicted fractal globular model (*s*^−1^, Fig. 4a), while chromatin interaction frequency of in situ Hi-C decayed at a rate closer to *s*^−0.5^ within the genomic distance of 1 Mb (Fig. 4a). The relative chromatin interaction frequency of in situ Hi-C was only about the half of SAFE Hi-C at 10 kb, which went up stably and became higher than SAFE Hi-C around 140 kb and continually rose higher as genomic distance increased (Fig. 4b).

This comparison revealed an unexpected bias in Hi-C library amplification, which could be due to the competition between the hybridization of complementary fragments and the primer annealing to target fragments. We speculate that ligates of high concentration tend to hybridize within the complementary DNA chains of their own instead of hybridizing with primers.

### SAFE Hi-C maintains high contact frequency in human genomic TADs

So far, TADs, compartments, and loops have been characterized using data from amplified Hi-Cs. The amplification effects on such analysis had not been evaluated experimentally. With the development of SAFE Hi-C, we were able to determine if and to what extent amplification affects TADs, compartments, and loops analysis.

We first plotted heatmaps of *Drosophila* S2 cells using normalized datasets and characterized TADs at 5 kb resolution (Fig. 3c and Supplementary Fig. 5). Overall, we observed minimal variation in border strength across the genome after amplification (Fig. 3c). Consistently, the number of identified TADs did not change much and most TADs were conserved (Fig. 3d). These results suggest that SAFE Hi-C is at least as reliable as traditional in situ Hi-C for TAD characterization for *Drosophila* genome.

We further calculated the interaction frequency vs. distance within TADs (Fig. 3e). Similar to the global decaying pattern, normalization against SAFE Hi-C within TADs revealed that chromatin interaction frequency was underrepresented within 3 kb and over-represented beyond 3 kb in most amplified libraries (Fig. 3f). Recently, sub-kb resolution Hi-C identified 4123 TADs in the *Drosophila* genome, with TADs as small as 3 kb^25^. Consistent with that, our SAFE Hi-C results also showed high frequency of interactions within a 3 kb range, which could be an important feature of the *Drosophila* genome.

Next, we identified TADs for SAFE Hi-C and in situ Hi-C conducted on human K562 cells. Most of the TADs identified overlapped (2946); however, more SAFE Hi-C-specific TADs (1668) were identified than in situ Hi-C-specific ones (556) (Fig. 4c). Visual inspection revealed that the fluctuation patterns of border index value were quite similar for SAFE and in situ Hi-Cs (Fig. 4d). However, the border index values of in situ Hi-C were overall lower (Fig. 4d) and the shape of TADs was generally fuzzier than those of SAFE Hi-C (Fig. 4d). We aligned the borders of all identified TADs, shared TADs and specific TADs for SAFE Hi-C and in situ Hi-C, and calculated the ratio of chromatin interaction frequency between intra- and inter-TADs (Fig. 4e). The ratios were consistently higher in the SAFE Hi-C (15.55, 15.07, and 15.58) than in the in situ Hi-C (9.26, 8.49, and 8.81) (Fig. 4e). These results together suggest that amplification weakens the intra-TAD contact probability but enhances the inter-TADs interaction chance, which could be caused by elevated amplification of ligates of fragments separated by longer genomic distance as shown in Fig. 4b. These observations further underline the importance of omitting amplification to improve the quality of Hi-C analysis.

### SAFE Hi-C reveals local chromatin structure of human *β-globin* locus

**Fig. 5:**
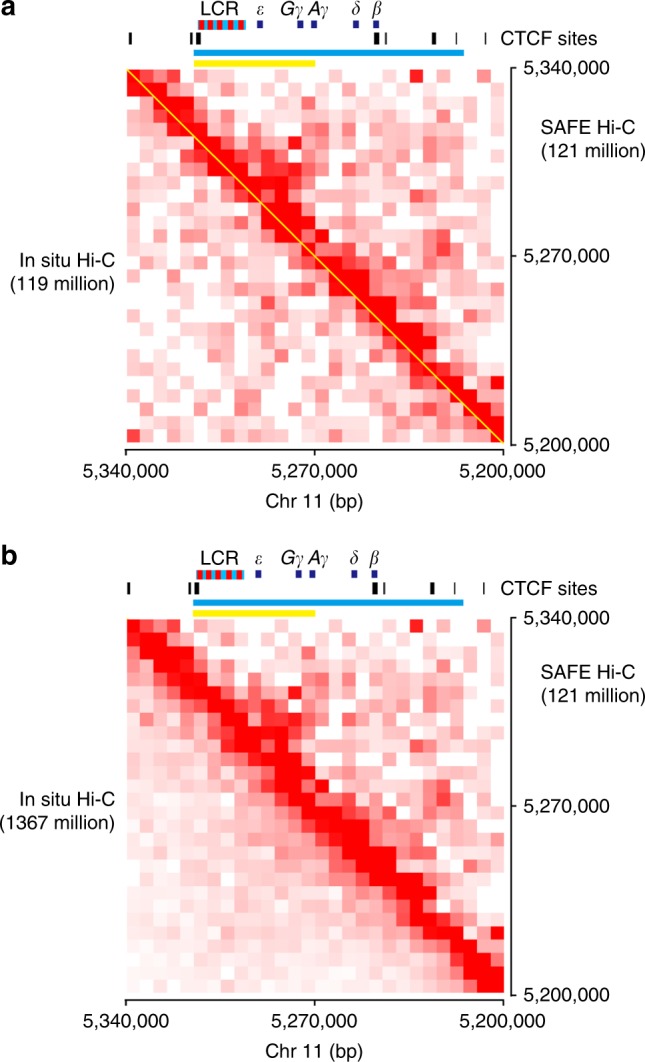
Local chromatin structure of the human *β-globin* locus. **a** Side-by-side comparison of chromatin structure of the human *β-globin* locus. Paired reads from similar sequencing depth of SAFE (*s*implified, *a*mplification-*f*ree, and economically *e*fficient process) Hi-C (121 million) and in situ Hi-C (119 million) were used for heatmap plotting. Human *β-globin* locus is shown at the top with hypersensitive sites in red and globin genes in dark blue rectangles, respectively. CTCF binding sites are shown as black vertical lines below *β-globin* locus. Yellow line and blue line correspond to genomic regions of small and large domain identifiable in SAFE Hi-C heatmap. The genomic region depicted here is from 5.20 to 5.34 Mb on human chromosome 11 spanning across the *β-globin* locus. **b** Side-by-side comparison of chromatin structure at human *β-globin* locus. Sequencing depth of in situ Hi-C is about 11 times more than that in **a**

The transcriptional regulation of human *β-globin* locus had been intensively studied. Hypersensitive sites in the locus control region interact with the downstream target genes of *ε-*, ^*G*^*γ-*, and ^*A*^*γ-globin* and activate their expression in K562 cell. To compare if SAFE Hi-C and in situ Hi-C differ in their ability of revealing the local 3D structure of this locus, we plotted heatmaps spanning 5.20–5.34 Mb on human chromosome 11 where the whole *β-globin* locus resides. Interestingly, two domains were visually identified in SAFE Hi-C heatmap, a small domain spanning across a region from hypersensitive site 5 (HS5) to ^*A*^*γ* gene and a big domain covering more sequences from HS5 to the 3′hypersensitive site 1 (3′HS1) and another CTCF binding site downstream (Fig. 5a). The existence of the small and big domains is consistent with current understanding of the domain formation by active genes and the border formation by CTCF-mediated looping, respectively. In contrary to SAFE Hi-C, on the heatmap plotted with paired reads from in situ Hi-C of similar sequencing depth (119 million), neither the small nor the big domain could be identified by visual inspection (Fig. 5a). We further plotted heatmap of in situ Hi-C with 11 times more paired reads. However, domain structures were still not recognizable (Fig. 5b). These results suggest that SAFE Hi-C is more sensitive than in situ Hi-C in revealing fine chromatin architecture even at much lower sequencing depth.

### Amplification effects on compartment and chromatin loop analysis

We characterized compartments for *Drosophila* (Supplementary Fig. 6) and human genomes. For both *Drosophila* S2 and human K562 cells, the eigenvalues correlated well between SAFE Hi-C and in situ Hi-C (Supplementary Figs. 7 and 8). Together, these results show that the effect of low amplification cycles on compartment analysis is less obvious than on TADs identification.

Finally, we identified long-range chromatin interactions at 5 kb resolution for the *Drosophila* genome (*q* value <0.1), but not for human genome because whose resolution was too low for meaningful and reliable chromatin loop identification. Signal-to-noise ratio was calculated as described^5^. Number of identified loops negatively correlated with amplification cycle (Supplementary Figs. 9 and 10a). About 43% of loops from each amplified Hi-C library (Supplementary Fig. 10a, dark blue bar) overlapped with those identified for SAFE Hi-C. The values of the Peak to Lower Left (P2LL)^5^ and *Z*-score Lower Left (*Z*scoreLL)^5^ of aggregated peak changed little for both SAFE Hi-C and amplified libraries (Supplementary Fig. 10b, c). For shared loops, the P2LL values increased as more PCR cycles repeated (Supplementary Fig. 10d), suggesting an over-amplification of these interactions happened. However, for loops lost after amplification, the P2LL values decreased (Supplementary Fig. 10d), suggesting an under-amplification of these interactions occurred.

## Discussion

In addition to the introduction of PCR duplicates and a dramatic reduction in library complexity, amplification has different effects on the three-dimensional genome architecture analysis for small (*Drosophila*) and big (human) genomes. For a small genome of *Drosophila*, amplification seems to compromise the characterization of loops and, to a lesser extent, of compartments, and amplification affects little on TAD identification. Differently, amplification compromises the TAD analysis more severely for human genome.

We tested the number limit of human cell for SAFE Hi-C. With 250 thousand of K562 cells, we successfully recovered an amount of ligates enough for sequencing in one-fourth lane on the Illumina HiSeq X10 platform. With a lower number of cells, the chance of DNA loss increased during library preparation. Although 250 thousand human cells were enough for SAFE Hi-C, 1–2 million mammalian cells will be easier to work with. Theoretically, there could be hundreds of billions of unique ligates in a SAFE Hi-C library prepared from 1–2 million mouse or human cells, a complexity not easy to reach with other Hi-C methods.

The better performance of SAFE Hi-C compared to in situ Hi-C is largely lying in its abilities of maintaining the original complexity of chromatin interactions, effectively lowering sequencing depth and saving labor and cost. In sum, by avoiding amplification, SAFE Hi-C can be used to improve the quality of Hi-C analysis as well as to save time, reagents, and to reduce cost. In case the availability of cell is a problem, PCR amplification can also be used after the finishing of SAFE Hi-C, so enough DNA material can be produced for high-throughput sequencing. Furthermore, other enzymes like DNase or MNase can also be used for SAFE Hi-C if the procedures are modified properly.

## Methods

### Cell culture

S2 cells were cultured in Schneider’s medium (Gibco, 21720024) supplemented with 10% heat-inactivated fetal bovine serum (Sigma, F7524) and 1% penicillin/streptomycin (Sigma, P0781) at 27 °C.

K562 cells were incubated in 1× RPMI1640 media supplemented with 10% fetal bovine serum at 37 °C with 5% CO_2_.

### In situ Hi-C

In situ Hi-C was carried out as described^5^. Cells were crosslinked with 1% formaldehyde then lysed to collect nuclei. Pelleted nuclei were digested with *Dpn*II restriction enzyme (NEB, R0147). The restriction fragment overhangs were filled and marked with biotin-labelled dATP (Thermo Fisher, 19524016) and dCTP, dTTP, and dGTP before ligation. DNA was reverse crosslinked, purified, and fragmented by sonication on a Covaris sonicator. Biotin-labelled DNA was pulled-down on Streptavidin Dynabeads (NEB, S1420S). After DNA repair and 3′ A addition, SHORT Y-Adaptor (Supplementary Table 1) was added. Diluted DNA on Dynabeads was used for PCR amplification (4, 8, 12,16, and 20 cycles) to produce similar amounts of DNA for sequencing on the Illumina HiSeq X10 platform (paired end 2 × 150 bp reads).

### SAFE Hi-C

SAFE Hi-C is a modification of in situ Hi-C^5,26^. Cells were crosslinked with 1% formaldehyde for 10 min at room temperature. The reaction was stopped by adding 1/10 volume of 2.5 M glycine. Up to 30 million crosslinked cells were resuspended in 500 µL of ice-cold Hi-C lysis buffer and rotated at 4 °C for 30 min. Nuclei were pelleted at 4 °C for 5 min at 2500 relative centrifugal force, and the supernatant was discarded. Pelleted nuclei were washed once with 500 µL of ice-cold Hi-C lysis buffer. The supernatant was removed again, and the pellet was resuspended in 100 µL of 0.5% sodium dodecyl sulfate (SDS) and incubated at 62 °C for 10 min with no shaking or rotation. Two hundred and eighty five microliters of water and 50 µL of 10% Triton X-100 were added, and samples were rotated at 37 °C for 15 min to quench the SDS. Fifty mircroliters of NEB buffer 3.1 and 20 µL of 10 U/µL *Dpn*II restriction enzyme (NEB, R0147) were then added, and the sample was rotated at 37 °C for 4 h. *Dpn*II was then heat inactivated at 62 °C for 20 min with no shaking or rotation. To fill in the restriction fragment overhangs and mark the DNA ends with biotin, 52 µL of incorporation master mix was then added: 37.5 µL of 0.4 mM biotin-dATP (Thermo Fisher, 19524016); 4.5 µL of dCTP, dGTP, and dTTP mix at 10 mM each; and 10 µL of 5 U/µL DNA Polymerase I Large (Klenow) Fragment (NEB, M0210). The reactions were then rotated at 37 °C for 45 min. Nine hundred and forty-eight microliters of ligation master mix was then added: 150 µL of 10× NEB T4 DNA ligase buffer with 10 mM ATP (NEB, B0202), 125 µL of 10% Triton X-100, 3 µL of 50 mg/mL bovine serum albumin (Thermo Fisher, AM2616), 10 µL of 400 U/µL T4 DNA ligase (NEB, M0202), and 660 µL of water. The reactions were then rotated at 16 °C for 4 h and room temperature for 1 h. Forty five microliters of 10% SDS and 55 µL of 20 mg/mL proteinase K were added for crosslinking reversal. Incubate at 55 °C for at least 2 h (overnight recommended). DNA was purified by phenol:chloroform:isoamyl alcohol (25:24:1) extraction. Purified DNA in solution was transferred into a 1.5 mL tube and sonicated to 400 bp on a Covaris sonicator. Biotin-labelled DNA was pulled-down on Streptavidin Dynabeads (NEB, S1420S). After DNA repair and 3′ A addition, Full Y-Adaptor (Supplementary Table 1) was added. DNA-on Dynabeads was resuspended in 100 μL of 0.8× PCR buffer and incubated at 98 °C for 10 min before being cooled off in ice water. The supernatant was recovered, quantified, and used for direct sequencing on the Illumina HiSeq X10 platform (paired end 2 × 150 bp reads).

SAFE Hi-C on human K562 cells was carried out similarly with the reagents reduced in proportion to the estimated chromatin contents, not the cell number.

### Data processing

We chose *Drosophila* dm3 and human hg19 versions of reference genome to align sequenced reads. Mapping, filtration, duplication removal, construction, and normalization of contact matrices and basic library statistics of reads from all experiments were processed using the Juicer pipeline^27^. For SAFE Hi-C, the analysis should only remove optical duplicates, which is caused by sequencing when a single cluster of reads is part of two adjacent tiles’ on the same slide and used to compute two read calls separately. We used a modified AWK script derived from juicer’s dups.awk script, which removes duplicates and judges the source of duplicates (PCR or optical) to remove only optical duplicates. We set 1 as map quality threshold and all downstream analyses were based on KR normalized matrices^24^, which ensures that each row and column of the contact matrix sums to the same value.

### Analysis of PCR duplication rates for *Drosophila* S2 libraries

For duplication depth analysis, we used a modified AWK script derived from juicer’s dups.awk script to count the duplicate number of each duplicated contact. We also tested different wobble number (0, 1, 2, and 3) to process the deduplication step for each library. We showed that SAFE Hi-C libraries had high library complexity in standard deduplication (wobble = 4) process and even higher when set wobble to 0.

### Library complexity estimation for *Drosophila* S2 libraries

Estimation of library complexity has been described before^5,28^. For SAFE Hi-C libraries, we computed the PCR duplication rate (although this library does not contain real PCR duplicates) to estimate library complexity.

### Topologically associating domain identification

Identification of TADs in *Drosophila* S2 cells was processed by Juicer Arrowhead algorithm at 5 kb resolution. For the identification of TADs in human K562 cells, border strength index was calculated at 25 kb resolution with a moving block size of 8 bins. TAD borders of human K562 dataset were defined by calling peaks through R package pracma. We used bedtools^29^ intersect command to call overlapped TADs and the overlapped region between two overlapped TADs should span at least 90% of each TAD range (command: bedtools intersect -f 0.9 -F 0.9 -sortout -a $tad_1 -b $tad_2).

### Compartment analysis

Method of compartment analysis has been described before^6^. We used the Pearson’s and eigenvector command of Juicer tools to obtain the Pearson’s correlation matrix and eigenvector at 10 kb resolution.

### Long-range chromatin interaction calling and aggregate peak analysis

Loops were identified at 5 and 10 kb resolution, respectively, using Juicer’s HiCCUPs algorithm^27^ (parameters: -m 2048 -r 5000,10000 -k KR–ignore_sparsity). P2LL and *Z*scoreLL were defined to measure the enrichment of HiCCUPs peaks during aggregate peak analysis^5^. P2LL is the ratio of the central pixel to the mean of the pixels in the lower left corner. *Z*scoreLL stands for the *Z*-score of the central pixel relative to all of the pixels in the lower left corner. Note that, for P2LL scatter plot (Supplementary Fig. 10d), the P2LL value is the ratio of each peaks’ pixel to the expect value of lower left.

### Statistics and reproducibility

We used R for statistical analysis.

Pythod package Matplotlib and basic graphics function in R were used to generate most of the figures. For Venn diagram of overlap of TAD borders, we utilized R package Venn Diagram. R package Matrix was used for operation of Hi-C sparse matrix. We set parameter minpeakdistance = 10 in function findpeaks of R package pracma for identification of TAD borders based on border strength. A modified version of pygenomictracks^30^ was used to plot Fig. 5 and Supplementary Fig. 9. SCCs of Supplementary Fig. 3 were calculated by R package HiCRep^31^ using 100 kb resolution matrix.

### Reporting summary

Further information on research design is available in the Nature Research Reporting Summary linked to this article.

## Supplementary information

Supplementary Information

Description of Additional Supplementary Files

Supplementary Data 1

Reporting Summary

## Data Availability

The Gene Expression Omnibus (GEO) accession code for the *Drosophila* S2 raw Illumina reads analyzed in this paper is PRJNA470784. The GEO accession code for the human K562 raw Illumina reads is PRJNA524051. Source data for the figures presented in this article are available in Supplementary Data 1. All other data reported in this paper are available upon request from the corresponding author.

## Code availability

All custom codes are available at http://github.com/shenscore/Safe_Hi-C_script/.
