# Supplementary material for: Amplification-free library preparation with SAFE Hi-C uses ligation products for deep sequencing to improve traditional Hi-C analysis
Source: Commun Biol. 2019 Jul 19;2:267. doi: 10.1038/s42003-019-0519-y (PMC6642088; doi:10.1038/s42003-019-0519-y)
Supplement: Supplementary file 1 — Supplementary Information [file 42003_2019_519_MOESM1_ESM.pdf]

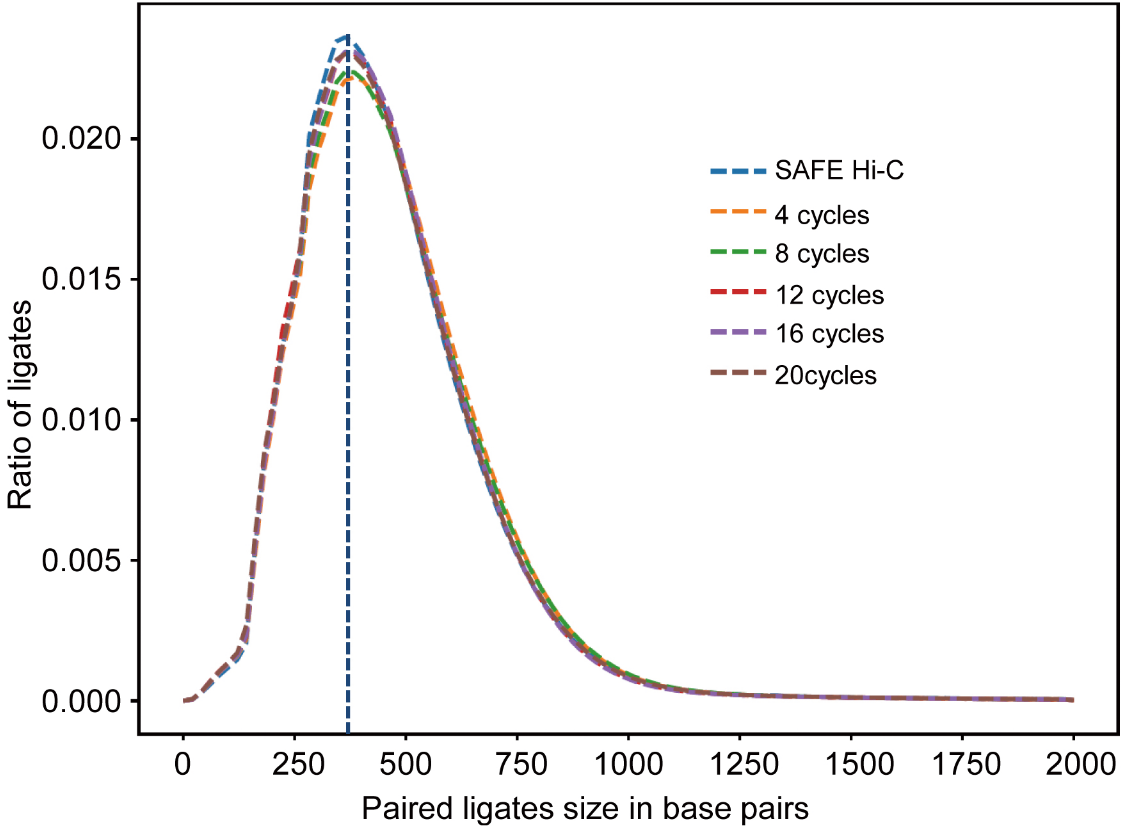

Supplementary Fig. 1. Size distribution of ligation products from Hi-Cs on *Drosophila* S2. Size was calculated based on the distance of each end of a paired read to the closest DpnII site.

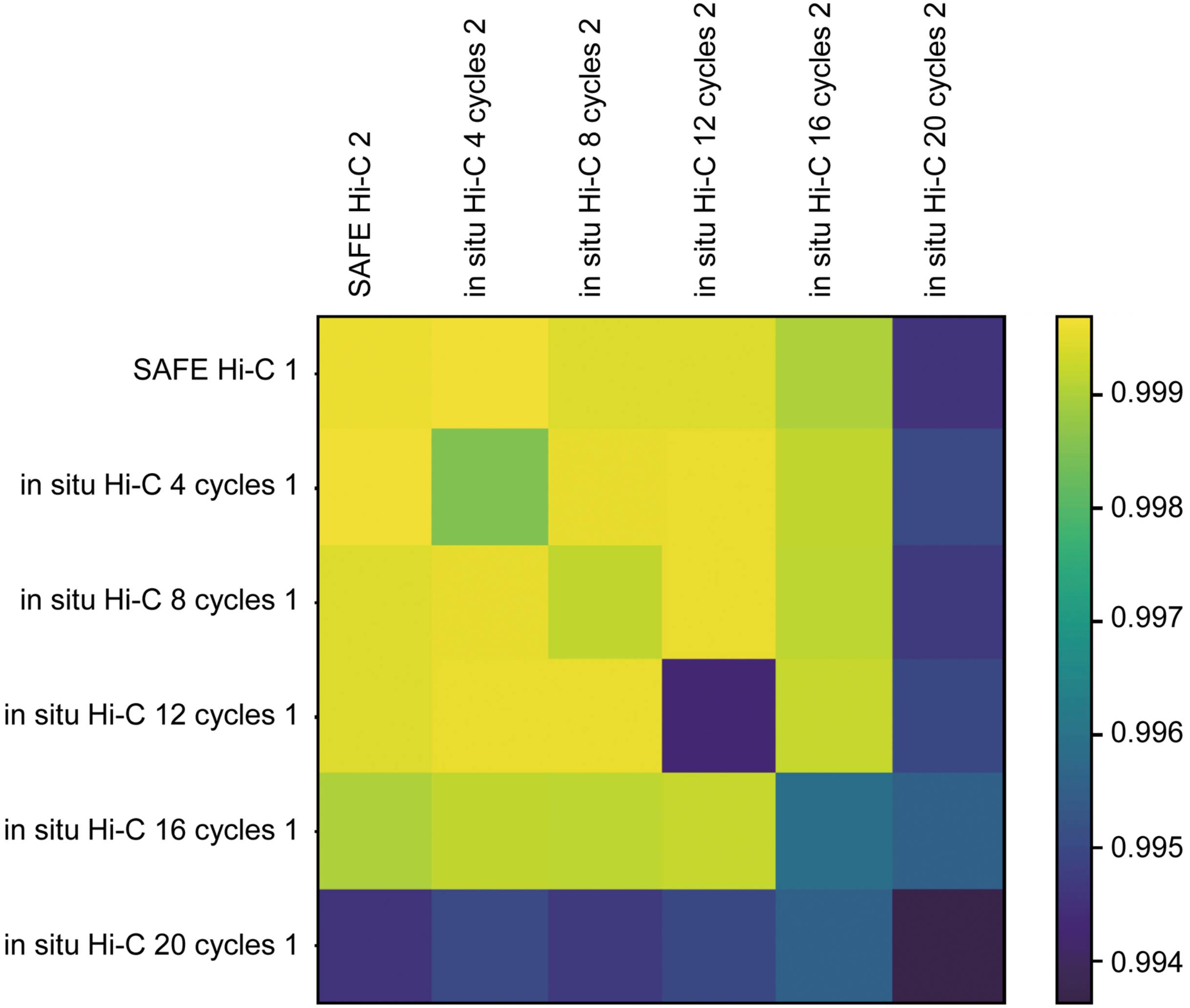

Supplementary Fig. 2. Stratum-adjusted correlation coefficients for biological duplicates of SAFE Hi-C and *in situ* Hi-C libraries amplified by different number of cycles for *Drosophila* S2.

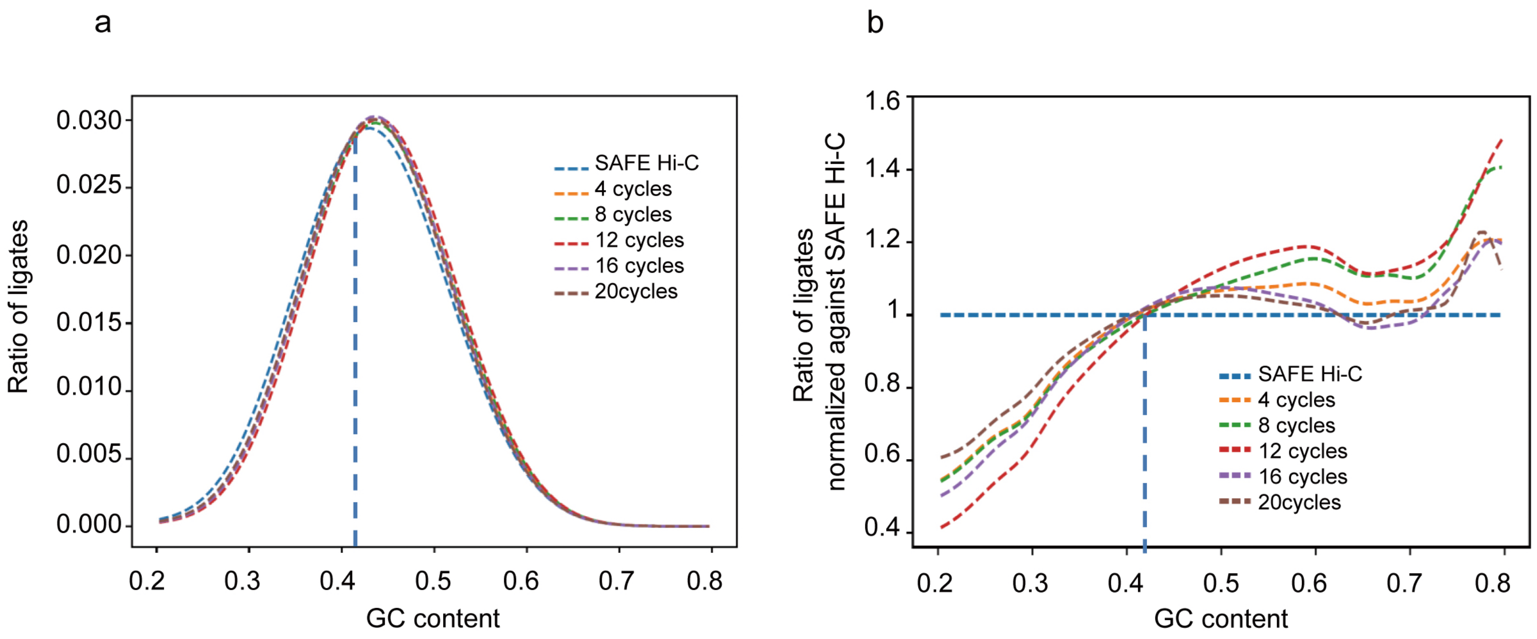

Supplementary Fig. 3. Amplification effect on paired reads of different GC content.

Paired reads with GC content from 20%-80% were equally divided into 100 equal bins.

(a). Ratio of paired reads as a function of GC content for SAFE Hi-C and in situ Hi-C libraries amplified for different number of cycles. Ratio is the number of paired reads over the number of total paired reads in each specific bin.

(b). Ratio of paired reads was normalized against SAFE Hi-C.

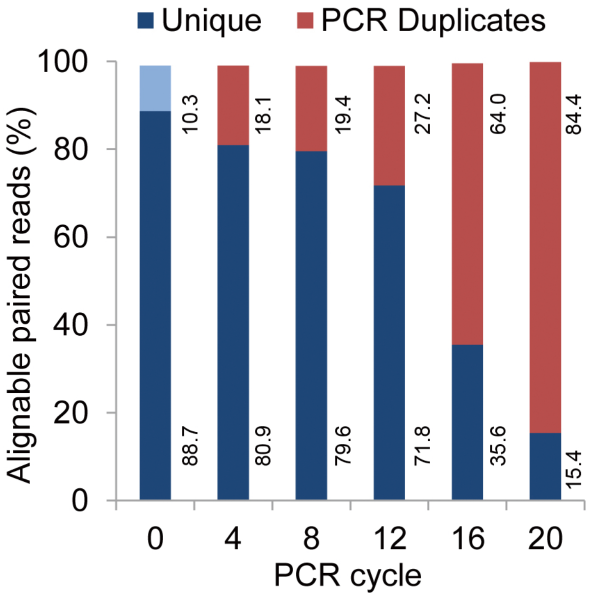

Supplementary Fig. 4. Percentage of unique paired reads and duplicates of the total alignable paired reads. The light blue bar shows the duplicates in SAFE Hi-C library that we kept.

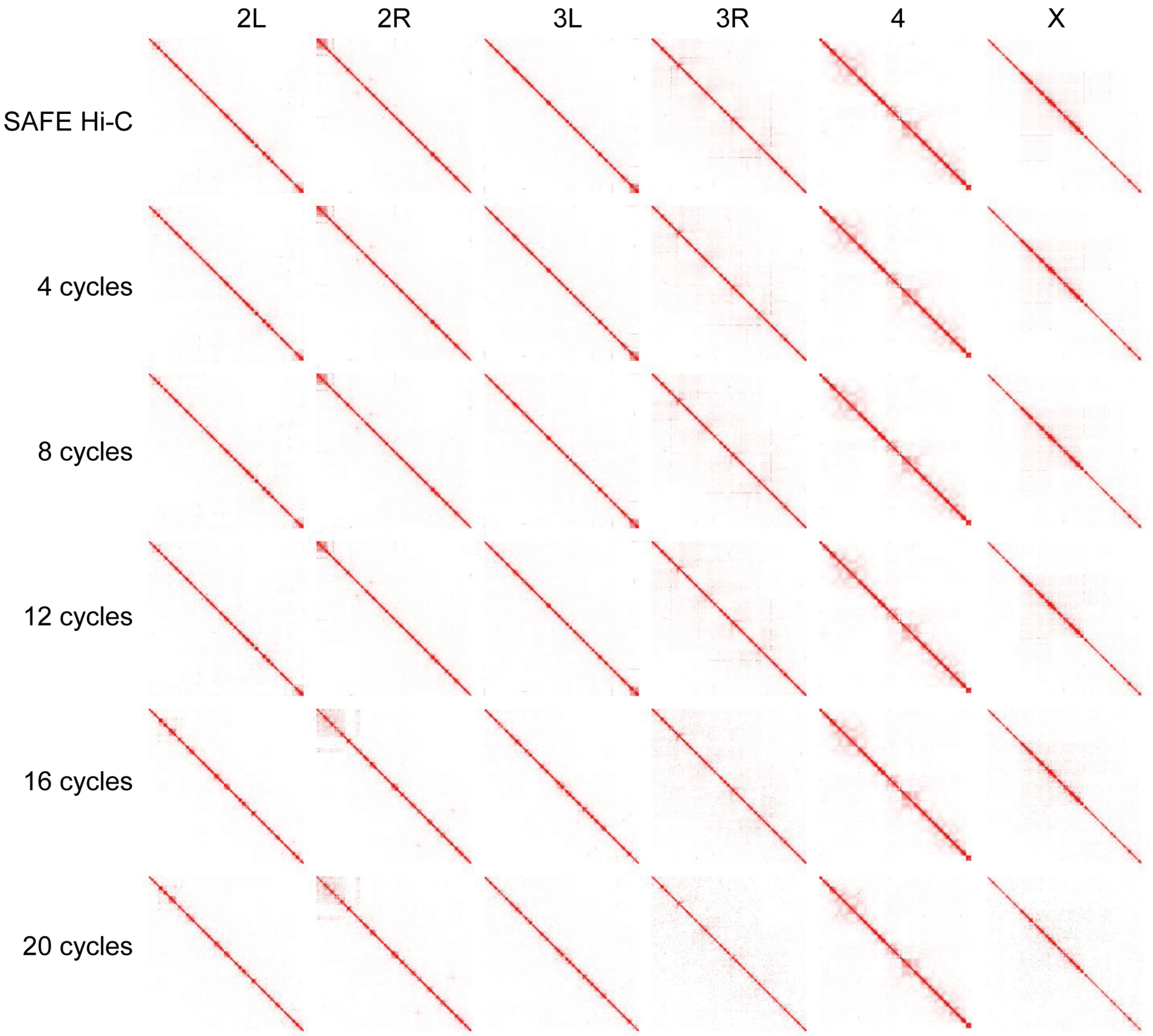

Supplementary Fig. 5. 5 kb resolution heatmaps for each chromosome arm of *Drosophila* S2 genome.

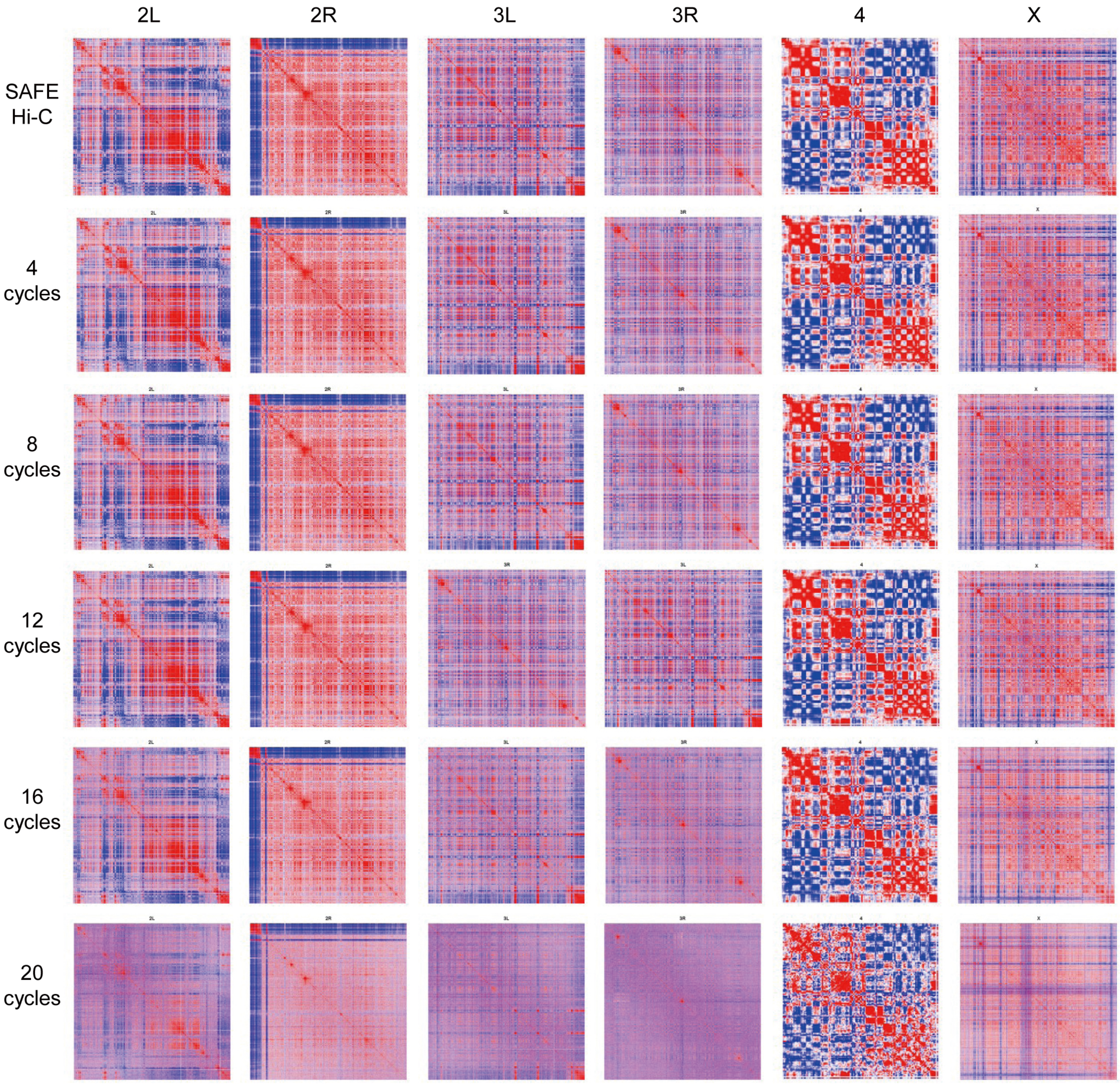

Supplementary Fig. 6. 10 kb resolution compartments for each chromosome arm of *Drosophila S2* genome.

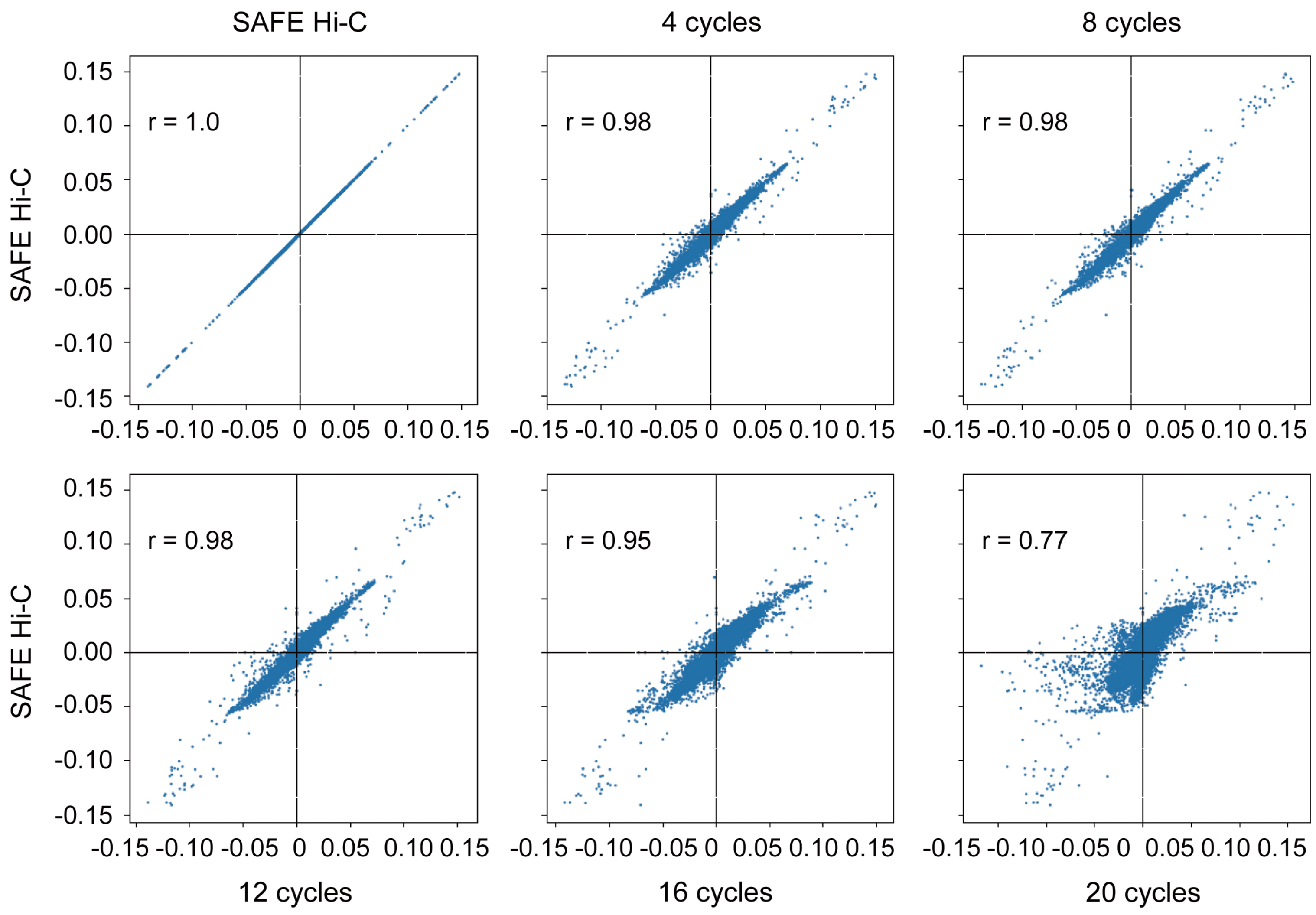

Supplementary Fig. 7. Pearson's correlation analysis of compartment eigenvalues of SAFE Hi-C and amplified Hi-Cs for *Drosophila* S2 cells.

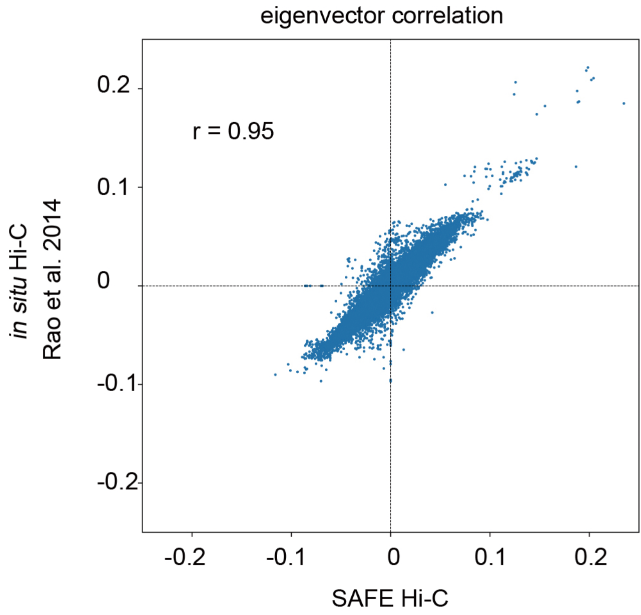

Supplementary Fig. 8. Pearson's correlation analysis of compartment eigenvalues of SAFE Hi-C and in situ Hi-C for human K562 cells.

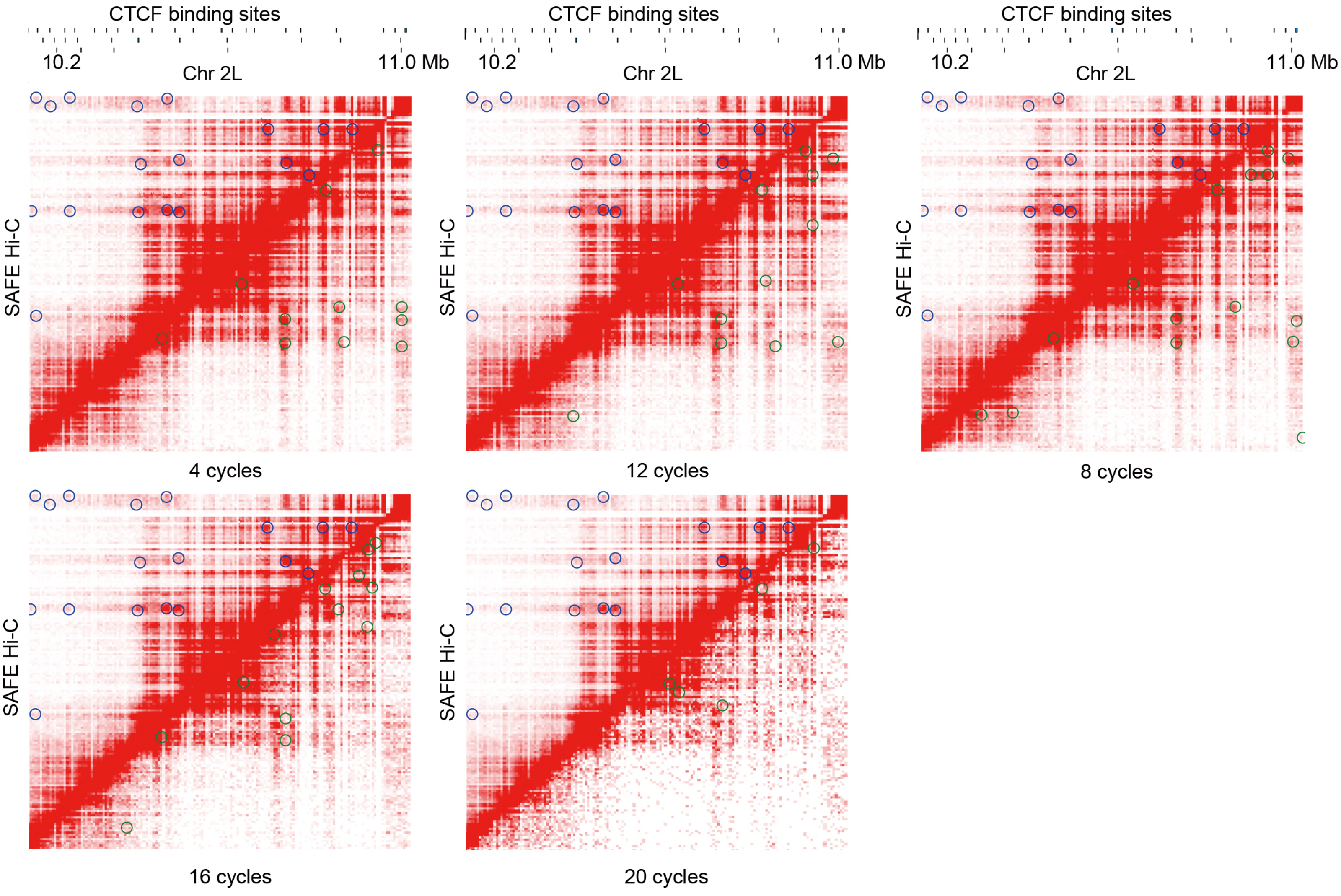

Supplementary Fig. 9. Examples of long-range chromatin loops identified in the region between 10.2Mb–11.0 Mb on the Chr 2L of the *Drosophila* genome by SAFE Hi-C and amplified Hi-Cs. Loops identified for SAFE Hi-C and amplified Hi-Cs are shown in blue and green circles, respectively. Short vertical lines above heatmaps indicate CTCF binding sites. *Drosophila* S2 CTCF binding sites were downloaded from modENCODE.

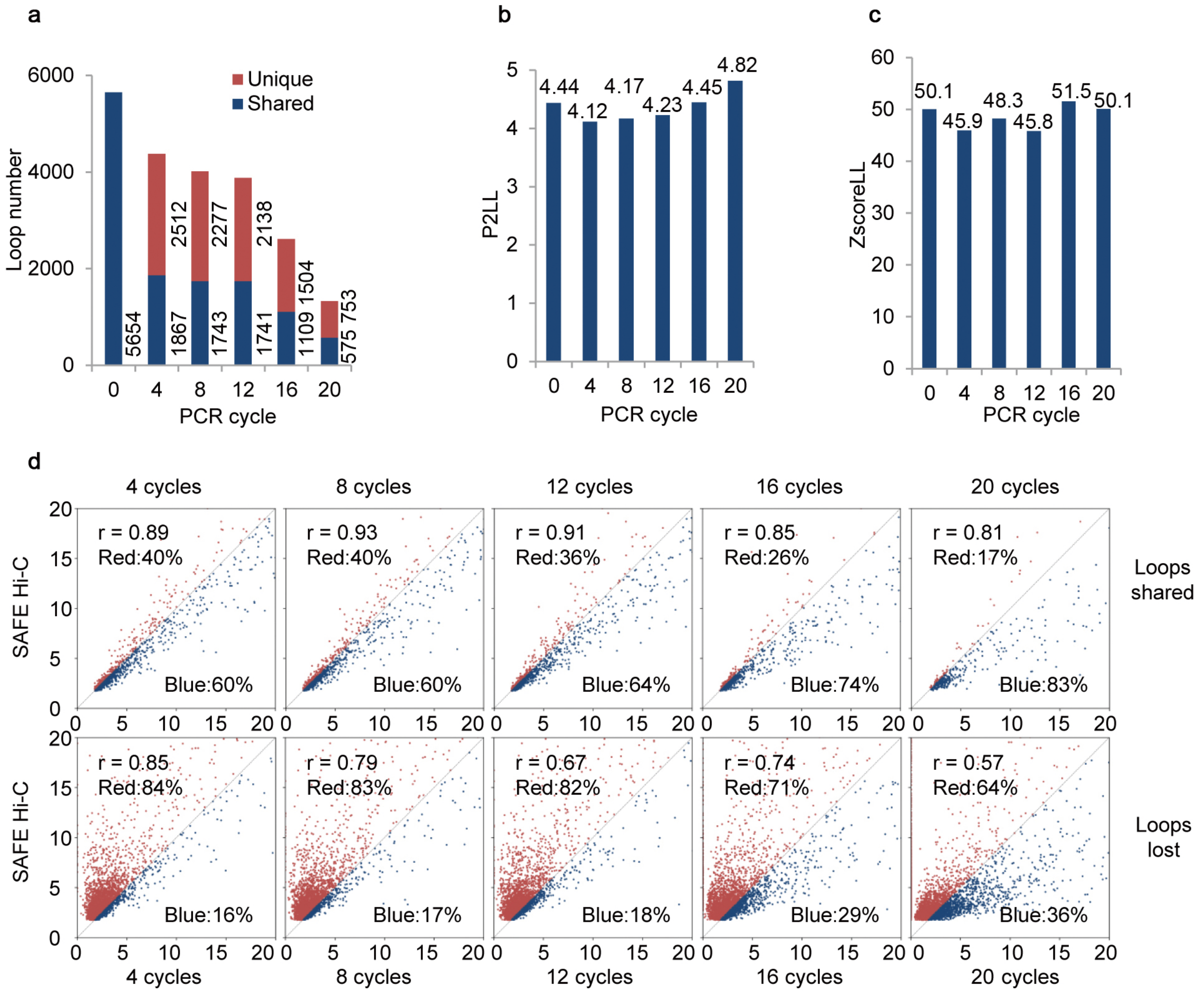

Supplementary Fig. 10. Comparison of long-range chromatin interactions loops identified in the *Drosophila* genome by SAFE Hi-C and amplified libraries.

(a). Number of long-range chromatin interaction loops. Dark blue bars show the number of loops shared with SAFE Hi-C, red bars show the number of loops uniquely found for amplified Hi-Cs.

(b). Average signal-to-noise values. P2LL was calculated as described1.

(c). Average ZscoreLL for identified chromatin loops. ZscoreLL was calculated as described1.

(d). P2LL correlation between loops shared and loops found in SAFE Hi-C but lost after amplification. Red and blue dots show loops with increased or reduced P2LL values after amplification.

**SHORT Y-Adaptor:**	
sY-Adaptor-F:	5'piGATCGGAAGAGCACACGTCTGAACTCCAGTCAC3'
sY-Adaptor-R:	5'TACACTCTTTCCCTACACGACGCTCTTCCGATCT3'
**FULL Y-Adaptor:**	
fY-Adaptor-F:	5'AATGATACGGCGACCACCGAGATCTACACTCTTTCCCTACACGACGCTCTTCCGATCT3'
fY-Adaptor-R:	5'GATCGGAAGAGCACACGTCTGAACTCCAGTCACATCACGATCTCGTATGCCGTCTTCTGCTTG3'
**PCR primer:**	
Universal primer 1.0:	5'AATGATACGGCGACCACCGAGATCTACACTCTTTCCCTACACGAC3'
Index primer 1.0:	5'CAAGCAGAAGACGGCATACGAGAT/CGTGATGT/GACTGGAGTTCAGACGT3'

Supplementary Table 1. List of adaptors and primer sequences.

	SAFER Hi-C	in situ Hi-C 4 cycles	in situ Hi-C 8 cycles	in situ Hi-C 12 cycles	in situ Hi-C 16 cycles	in situ Hi-C 20 cycles
Sequenced Read Pairs	443,161,104	322,561,216	290,181,793	305,911,699	311,416,275	325,737,701
Normal Paired	190,828,243	139,965,592	124,672,768	128,887,142	132,354,738	136,207,715
Chimeric Paired	147,261,678	105,612,129	95,477,326	103,429,671	105,371,575	111,731,045
Chimeric Ambiguous	79,030,016	59,772,203	54,624,219	58,129,188	57,940,532	61,604,888
Unmapped	26,041,167	17,211,292	15,407,480	15,465,698	15,749,430	16,194,053
Ligation Motif Present	338,736,272	247,368,370	223,871,283	240,316,348	243,927,225	258,406,662
Alignable	338,089,921	245,577,721	220,150,094	232,316,813	237,726,313	247,938,760
Unique Reads	299,929,328	198,866,672	175,272,581	166,734,491	84,573,363	38,180,490
PCR Duplicates	35,059,438	44,414,379	42,724,700	63,300,068	152,094,594	209,367,729
Optical Duplicates	3101155	2,296,670	2,152,813	2,282,254	1,058,356	390,541
Library Complexity Estimate	1484609396	581922523	480376696	336812519	91405541	38229560
Intra-fragment Reads	8,188,657(2.44%)	2,992,149(1.61%)	2,668,207(1.63%)	2,478,636(1.59%)	1,071,523(1.35%)	348,989(0.97%)
Below MAPQ Threshold	81,242,399(24.25%)	45,850,518(24.68%)	40,713,164(24.85%)	41,046,284(26.40%)	34,234,664(43.09%)	23,081,100(64.30%)
Hi-C Contacts	245,557,710(73.30%)	136,912,869(73.71%)	120,467,844(73.52%)	111,942,174(72.00%)	44,151,206(55.57%)	12,467,140(34.73%)
Ligation Motif Present	131,525,095 (39.26%)	69,971,976 (37.67%)	62,100,601 (37.90%)	58,626,298 (37.71%)	23,101,358 (29.07%)	6,361,221 (17.72%)
3' Bias (Long Range)	70% - 30%	69% - 31%	69% - 31%	70% - 30%	70% - 30%	70% - 30%
Pair Type %(L-I-O-R)	25%-25%-25%-25%	25%-25%-25%-25%	25%-25%-25%-25%	25%-25%-25%-25%	25%-25%-25%-25%	25%-25%-25%-25%
Inter-chromosomal	16,991,943 (5.07%)	9,645,911 (5.19%)	8,451,464 (5.16%)	7,816,689 (5.03%)	3,111,425 (3.92%)	909,767 (2.53%)
Intra-chromosomal	228,565,767 (68.23%)	127,266,958 (68.51%)	112,016,380 (68.37%)	104,125,485 (66.98%)	41,039,781 (51.65%)	11,557,373 (32.20%)
Short Range (<20Kb)	119,803,473 (35.76%)	62,999,574 (33.92%)	55,897,678 (34.12%)	52,279,252 (33.63%)	20,977,475 (26.40%)	6,029,361 (16.80%)
Long Range (>20Kb)	108,745,026 (32.46%)	64,258,102 (34.59%)	56,110,760 (34.25%)	51,838,856 (33.34%)	20,059,291 (25.25%)	5,527,110 (15.40%)

Supplementary Table 2. Libraries sequencing statistics for *Drosophila* S2 cells.

	SAFE Hi-C	*in situ* Hi-C replicates (*Rao et al.*)
Sequenced Read Pairs	120,626,961	118,785,918
Unmapped	710948 (0.59%)	6,608,116 (5.56%)
Ligation Motif Present	82791402 (68.63%)	24,214,726 (20.39%)
Alignable	106762759 (88.51%)	110,343,831 (92.89%)
Unique Reads	86056230 (71.34%)	109848235 (92.48%)
Intra-fragment Reads	1,539,033 (1.28%)	341,252 (0.29%)
Below MAPQ Threshold	7,896,573 (6.55%)	19,893,772 (16.75%)
Hi-C Contacts	105560358 (87.51%)	89,613,211 (75.44%)
Ligation Motif Present	34,795,109 (28.85%)	13,520,105 (11.38%)
3' Bias (Long Range)	69% - 31%	68% - 32%
Pair Type %(L-I-O-R)	25% - 25% - 25% - 25%	25% - 25% - 25% - 25%
Inter-chromosomal	11,756,385 (9.75%)	32,269,075 (27.17%)
Intra-chromosomal	64,864,239 (53.77%)	57,344,136 (48.28%)
Short Range (<20Kb)	23,669,261 (19.62%)	10,121,516 (8.52%)
Long Range (>20Kb)	41,194,674 (34.15%)	47,222,552 (39.75%)

Supplementary Table 3. SAFE Hi-C and *in situ* Hi-C sequencing statistics for human K562 cells.

Reference:

Rao, S. S. *et al.* A 3D map of the human genome at kilobase resolution reveals principles of chromatin looping. *Cell* **159**, 1665-1680, doi:10.1016/j.cell.2014.11.021 (2014).
