# Supplementary material for: Amplification-free library preparation with SAFE Hi-C uses ligation products for deep sequencing to improve traditional Hi-C analysis
Source: Commun Biol. 2019 Jul 19;2:267. doi: 10.1038/s42003-019-0519-y (PMC6642088; doi:10.1038/s42003-019-0519-y)
Supplement: Supplementary file 2 — Description of Additional Supplementary Files [file 42003_2019_519_MOESM2_ESM.docx]

**Description of Additional Supplementary Files**

**File Name**: Supplementary Data 1

**Description**: Source data for making graphs and charts in the main figures, specifically for Fig 2a, 2b, 2c, 2d and 2e;Fig 3a, 3b,3c,3d,3e and 3f; Fig 4a, 4b, 4c, 4d and 4e; Fig 5a and 5b.
