# Supplementary material for: Amplification-free library preparation with SAFE Hi-C uses ligation products for deep sequencing to improve traditional Hi-C analysis
Source: Commun Biol. 2019 Jul 19;2:267. doi: 10.1038/s42003-019-0519-y (PMC6642088; doi:10.1038/s42003-019-0519-y)
Supplement: Supplementary file 4 — Reporting Summary [file 42003_2019_519_MOESM4_ESM.pdf]

nature research

Corresponding author(s): Chunhui Hou

Last updated by author(s): Jun 4, 2019

Reporting Summary

Nature Research wishes to improve the reproducibility of the work that we publish. This form provides structure for consistency and transparency in reporting. For further information on Nature Research policies, see Authors & Referees and the Editorial Policy Checklist.

Statistics

For all statistical analyses, confirm that the following items are present in the figure legend, table legend, main text, or Methods section.

n/a	Confirmed	
☐	☒	The exact sample size (*n*) for each experimental group/condition, given as a discrete number and unit of measurement
☐	☒	A statement on whether measurements were taken from distinct samples or whether the same sample was measured repeatedly
☐	☒	The statistical test(s) used AND whether they are one- or two-sided<br>*Only common tests should be described solely by name; describe more complex techniques in the Methods section.*
☐	☒	A description of all covariates tested
☐	☒	A description of any assumptions or corrections, such as tests of normality and adjustment for multiple comparisons
☐	☒	A full description of the statistical parameters including central tendency (e.g. means) or other basic estimates (e.g. regression coefficient) AND variation (e.g. standard deviation) or associated estimates of uncertainty (e.g. confidence intervals)
☐	☒	For null hypothesis testing, the test statistic (e.g. *F*, *t*, *r*) with confidence intervals, effect sizes, degrees of freedom and *P* value noted<br>*Give P values as exact values whenever suitable.*
☒	☐	For Bayesian analysis, information on the choice of priors and Markov chain Monte Carlo settings
☒	☐	For hierarchical and complex designs, identification of the appropriate level for tests and full reporting of outcomes
☐	☒	Estimates of effect sizes (e.g. Cohen's *d*, Pearson's *r*), indicating how they were calculated

Our web collection on statistics for biologists contains articles on many of the points above.

Software and code

Policy information about availability of computer code

Data collection	Illumina BaseSpace Clarity LIMS
Data analysis	bwa (version 0.7.15); juicer (version 1.5); bedtools (version 2.27.1); parallel (version 20150322); R; AWK; python; maplotlib; scipy; excel

For manuscripts utilizing custom algorithms or software that are central to the research but not yet described in published literature, software must be made available to editors/reviewers. We strongly encourage code deposition in a community repository (e.g. GitHub). See the Nature Research guidelines for submitting code & software for further information.

Data

Policy information about availability of data

All manuscripts must include a data availability statement. This statement should provide the following information, where applicable:

- Accession codes, unique identifiers, or web links for publicly available datasets
- A list of figures that have associated raw data
- A description of any restrictions on data availability

The Gene Expression Omnibus (GEO) accession code for the Drosophila S2 raw Illumina reads analyzed in this paper is PRJNA470784). The GEO accession code for the human K562 raw Illumina reads is PRJNA524051. All data reported in this paper are available upon request from the corresponding author.

Field-specific reporting

Please select the one below that is the best fit for your research. If you are not sure, read the appropriate sections before making your selection.

☒ Life sciences ☐ Behavioural & social sciences ☐ Ecological, evolutionary & environmental sciences

Life sciences study design

All studies must disclose on these points even when the disclosure is negative.

Sample size	30 million Drosophila S2 cells and 250 thousand human K562 cells were used for each Hi-C experiment. Quantification of final DNA amount shows that this amount of cell is enough for amplification-free Hi-C.
Data exclusions	No data was excluded.
Replication	Hi-Cs were repeated in replicates. Pearson correlation analysis show very good reproducibility of our experiments.
Randomization	No randomization was carried out.
Blinding	Blinding is not relevant to this study as most of our data do not involve control vs. treatment.

Reporting for specific materials, systems and methods

We require information from authors about some types of materials, experimental systems and methods used in many studies. Here, indicate whether each material, system or method listed is relevant to your study. If you are not sure if a list item applies to your research, read the appropriate section before selecting a response.

Materials & experimental systems

n/a	Involved in the study	
☒	☐	Antibodies
☐	☒	Eukaryotic cell lines
☒	☐	Palaeontology
☒	☐	Animals and other organisms
☒	☐	Human research participants
☒	☐	Clinical data

Methods

n/a	Involved in the study	
☒	☐	ChIP-seq
☒	☐	Flow cytometry
☒	☐	MRI-based neuroimaging

Eukaryotic cell lines

Policy information about cell lines

Cell line source(s)	Drosophila S2 and human K562 cells
Authentication	S2 cells were obtained from DGRC and authenticated by growth in specific media, morphology and genomic DNA sequencing. K562 cells were authenticated by growth in specific medium, morphology and genomic DNA sequencing.
Mycoplasma contamination	PCR and genomic sequencing were performed to confirm cell are negative for mycoplasma contamination.
Commonly misidentified lines (See ICLAC register)	Drosophila S2 and human K562 cell lines are not in the database of commonly misidentified cell lines.
